# The Benefit of a Single Oral Dose of Ivermectin in Humans: The Adverse Effects on Cimex lectularius L. Populations and Fecundity

**DOI:** 10.7759/cureus.6098

**Published:** 2019-11-08

**Authors:** Johnathan M Sheele, Gale Ridge, Xiaolin Li, Danie Schlatzer, Elizabeth Lesser

**Affiliations:** 1 Emergency Medicine, Mayo Clinic, Jacksonville, USA; 2 Entomology, The Connecticut Agricultural Experimental Station, New Haven, USA; 3 Nutrition Proteomics and Small Molecule Mass Spectrometry, Case Western Reserve University, Cleveland, USA

**Keywords:** bed bug, bedbug, treatment, ivermectin, fecundity, reproduction, infestation, drug, cimex lectularius, cimicidae

## Abstract

Objective

To measure the population size and fecundity of the common bed bug Cimex lectularius L. (C. lectularius) after feeding it with the blood obtained from human subjects who have consumed a single dose of ivermectin.

Methods

Serial blood samples were obtained from two human subjects at hour 0 (control) and 4-96 hours after they received a single 0.2 mg/kg dose of ivermectin. The blood samples were then fed to 2,273 bed bugs. Bed bug incapacitation rates, fecundity, and population sizes were recorded over a 54-day period. Whole blood ivermectin levels were measured in the human subjects and the insects.

Results

The fold change in the size of the control group population over the course of the experiment was found to be 2.16. This was significantly greater (p: <.001) than for all the post-ivermectin feeding groups (range: -11.04-1.43). Two weeks after the experiment, the number of eggs laid per live adult female bed bug per day was 10.74 for controls, which was significantly different (p: <0.001) compared to all the post-ivermectin feeding groups (range: 0-4.28).

Conclusions

There were significant reductions in C. lectularius population size and fecundity in insects that fed on blood obtained from human study subjects up to 96 hours after they have consumed a single oral dose of ivermectin.

## Introduction

Cimex lectularius L. (C. lectularius), known as the common bed bug, is a pest and human ectoparasite [1]. One hospital reported finding bed bugs as frequently as every 2.2 days and, in the emergency department (ED), approximately every 4-5 days, resulting in significant institutional expense [2-5]. Survey and epidemiological studies suggest that many more patients with residential bed bug infestations are being seen by healthcare providers than the actual number of insects being identified by the healthcare providers [2,3,6-8]. The estimated number of ED visits related to bed bugs increased seven-fold between 2007 and 2010 [9]. ED patients with bed bug infestations are more likely to be older, male, arrive at the ED by ambulance, have higher emergency severity index (ESI) scores, and be admitted to the hospital [5]. There have not been any large-scale investigations exploring the bed bug microbiome, and bed bugs have not been shown to be a significant vector of human infectious diseases despite their ability to transmit some human pathogens in the laboratory [10-14]. Quantifying human morbidity associated with bed bugs requires additional investigation [1,14-17].

It has been estimated that bed bugs feed about every 2.5 days and that 40-90% of adult female bed bugs found in a harborage had fed in the past 24 hours [18]. The frequency with which bed bugs take blood meals provide an opportunity for humans to intervene using chemotherapeutic agents to manage infestations. The antiparasitic drug ivermectin has been shown to cause dose-dependent bed bug toxicity with adverse effects on fecundity, refeeding, mobility, and molting [19-22]. Most research on ivermectin and bed bugs have involved feeding bed bugs on blood samples spiked with ivermectin in the laboratory. However, feeding bed bugs in this manner cannot be directly correlated to the reported plasma ivermectin levels in humans for three reasons: 1) spiked blood samples in the laboratory will not account for ivermectin drug metabolites that have been previously suspected as having antiparasitic effects; 2) bed bugs take a whole blood meal, not just the plasma (the liquid portion of blood); and 3) plasma ivermectin levels do not account for any hematophagous intracellular drug accumulation that would be consumed by a feeding bed bug. The most effective way to study the toxic effects of ivermectin on bed bugs is by administering ivermectin to humans and having bed bugs serially feed on their natural hosts. There has only been one previous study involving bed bugs feeding on human blood after subjects were given oral ivermectin. It was limited by a low number of insects used and a short period of observation (20 days); and fecundity, an indicator of downstream harm, was not recorded [21].

Ivermectin is one of the most ubiquitous antiparasitic drugs used in medicine. It is administered to over 250 million people annually, most commonly in the tropical regions [23]. The drug is listed by the World Health Organization (WHO) as one of the essential medicines to combat numerous human parasites. A single oral 0.2 mg/kg dose in adults has an excellent safety profile, and the pharmacokinetics, pharmacology, and toxicity of ivermectin in humans are well known [23-28]. In two studies, fasting healthy human adult volunteers were given a single oral dose of 0.165 mg/kg of ivermectin. Blood plasma levels peaked at 46.6 (range: 16.4-101.1) and 30.6 (range: 13.9-68.4) ng/mL, respectively ~4 hours after ingestion [28]. Ivermectin acts on the invertebrate glutamate-gated chloride change, causing hyperpolarization of nerve and muscle cells [27].

This designed study sought to more efficiently evaluate the effects of ivermectin on a large population of bed bugs where ivermectin was orally administered to humans and bed bugs were serially fed on the corresponding blood samples and observed for 54 days. Additionally, we sought to correlate whole blood ivermectin levels in the study subjects and ivermectin levels in the bed bugs with the outcome data.

## Materials and methods

Human subjects

We obtained institutional review board approval from University Hospitals, Cleveland Medical Center, Cleveland, OH, to enroll two subjects between the ages of 18-50 years. We excluded persons who were pregnant or trying to get pregnant, drink more than two alcoholic beverages per day on average, used illicit drugs in the last month, had a known malignancy, had a known liver, kidney, neuromuscular, cardiac, or endocrine disease, had a known or suspected Loa loa filariasis, had taken ivermectin in the last month, or had allergies to ivermectin, soy, wheat, egg, or milk. Additionally, those persons taking warfarin, benzodiazepines, pentobarbital, sodium oxybate, valproic acid, amprenavir, rifampin, ritonavir, St. John’s wort, phenothiazines, amiodarone, erythromycin, verapamil, quinidine, ketoconazole, cyclosporine, tamoxifen, or carvedilol were excluded because of a suspected potential interaction with ivermectin.

Because high-fat meals may help with ivermectin absorption, our subjects received a meal consisting of 884 calories, 42 grams of fat, 410 mg of cholesterol, and 39 grams of protein prior to consuming a single 0.2 mg/kg dose of oral ivermectin [26]. Both subjects participated in the study at the same time and consumed their ivermectin concurrently. Subjects had 15 mL of blood drawn into ethylenediaminetetraacetic acid (EDTA) phlebotomy tubes prior to taking ivermectin (0 hour-control) and at 4, 12, 24, 36, 48, 60, 72, 84, and 96 hours after ivermectin consumption. For each blood draw, an aliquot of whole blood and plasma were frozen at -80 ‎°C, and the remainder of the blood sample was placed on ice until it was fed to a population of laboratory bed bugs. Subjects were paid USD 150 at the completion of the study.

Cimex lectularius L.

To limit human subjects’ exposure to the bed bugs, we chose to remove the subjects’ blood and feed it to the insects using an artificial feeding system as described previously [19,22].

Heterogenous inbreeding populations of different laboratory strains of C. lectularius were placed into 20 different 50 mL-test tubes prior to the first feeding. Each test tube contained a stiff piece of paper upon which the insects gathered. The cap for each test tube had a 1/2-inch hole drilled into it and a piece of sheer fabric glued to the surface. The insects were fed by placing a piece of parafilm over the cap and inverting the test tube into a petri dish containing warmed blood (40 °C). All bed bugs fed on subject blood within eight hours of it being drawn and each population of bed bugs only fed once on experimental blood. Unfed bed bugs were discarded, and only the 10 separate groups of fed C. lectularius were kept for observation.

All insects were offered an additional blood meal of previously frozen defibrinated sheep’s blood (HemoStat Laboratories, Dixon, CA) without ivermectin, using the previously described feeding techniques on days 21, 25, and 29. The experiment was stopped 54 days after the subjects consumed the ivermectin. Bed bugs from both subjects were combined for each time point for the data analysis.

Overall and female bed bug population sizes were recorded at the beginning and on day 54 after the ivermectin ingestion. The overall number of incapacitated bed bugs or those that were immobile or unable to remain attached to the paper perch inside the tube were counted on day 7, and overall mortality rates were calculated on day 7 and day 14 after the ivermectin ingestion. The number of incapacitated female bed bugs and the number of laid eggs were counted on day 14 after the ivermectin ingestion. We attempted to record the number of insects that refed on blood meals 3-4 weeks into the experiment but found it impossible to accurately identify new partially fed insects from those previously fed insects that had undigested blood meals. It has previously been shown that ivermectin causes bed bugs to incompletely digest their blood meals in a dose-dependent manner [19-22]. A small number of post-fed bed bugs were frozen at -80 °C to have ivermectin levels measured using high-performance liquid chromatography (HPLC) and mass spectroscopy (MS).

High-performance liquid chromatography (HPLC) and mass spectroscopy (MS): human whole blood

Ivermectin concentrations were analyzed using a modified method reported previously [29]. In summary, each sample was thawed and 50 µL of supernatant was mixed with 500 µL of 3:1 methanol:acetonitrile, vortexed for 20 seconds, and incubated at 4 °C for 30 minutes. The solution was centrifuged at 16,000 g for 20 minutes and 500 µL of supernatant was transferred to a clean tube and speed-vacuumed to dryness. It was then reconstituted with 50 µL of reconstitute solvent (0.5 mM ammonium formate, 0.1% formic acid in 50% methanol) and centrifuged at 16,000 g for 20 minutes. Five µL was used for HPLC and MS. The chromatography was performed with a reversed-phase C18 column (Atlantis dC18 column, 50 x 2.1 mm, 3 µm, Waters Corporation, Milford, MA). Ivermectin was separated from blood endogenous components using 10% 0.5 mM ammonium formate: 90% 0.1% formic acid in acetonitrile in isocratic mode at 0.2 mL/min. The column was set at 35 °C. Ivermectin was detected by a Thermo Scientific TSQ Quantum Ultra (Thermo Fisher Scientific, Waltham, MA) with Heated Electrospray Ionization (HESI-II, Thermo Fisher Scientific, Waltham, MA) probe using ESI positive ionization mode, spray voltage of 3,000 V, capillary temperature of 200 °C, vaporizer temperature of 300 °C, sheath gas pressure of 40, auxiliary gas pressure of 10, skimmer offset of 10 V, and with selected reaction monitoring (SRM) set up: Q1: 0.7 full width at half maximum (FWHM); Q3: 0.7 FWHM; Q2: 1.5 mTorr; scan width: 0.002 m/z, scan time at 0.02 seconds.

High-performance liquid chromatography and mass spectroscopy: Cimex lectularius L.

Frozen bed bugs were thawed to room temperature, weighed, 100 mL of 0.2% formic acid was added, and the sample was homogenized. Samples were then mixed with 1,000 mL of 3:1 v/v methanol:acetonitrile, vortexed for 20 seconds, sonicated for 10 minutes, and centrifuged at 16,000 g for 20 minutes. 1,000 mL of supernatant was transferred to a clean tube and speed-vacuumed to dryness. It was then reconstituted with 50 mL of reconstitute solvent (0.5 mM ammonium formate, 0.1% formic acid in 50% methanol) and then centrifuged at 16,000 g for 20 minutes. Five microliters of the supernatant were used for HPLC and MS. Ivermectin was detected and quantified using the same HPLC/MS conditions as for blood samples.

Data analysis

Continuous variables were summarized with median and range, unless otherwise specified, and categorical variables were summarized with frequency and percentage. Fold change in the number of overall healthy bed bugs and healthy female bed bugs from the beginning to the end of the experiment were evaluated using the Wilcoxon Rank Sum test between the control and post-ivermectin feeding groups. Proportional differences in incapacitation and mortality between post-ivermectin feeding groups and controls were assessed using Fisher’s Exact test. Single variable log-linear models were fit to estimate the effect of time from the original feeding on the number of overall alive bed bugs and the number of alive female bed bugs at the end of the experiment. Corresponding percentage change in the expected counts was calculated along with their 95% confidence intervals (CI). Single variable linear regression was used to estimate the relationship between the number of eggs laid per living adult female C. lectularius by the number of hours since the original feeding, excluding controls. Expected change in the number of eggs laid per living adult female and corresponding 95% CI were estimated. All tests were two-sided and a p-value of less than 0.05 was considered statistically significant.

## Results

There were 2,273 fed bed bugs in the experiment with the mean number of fed bed bugs for each group being 227.3 [standard deviation (SD) 56.6]. Table 1 summarizes the total change in bed bug population in each group 54 days after the experimental blood meal.

**Table 1:** Overall bed bug population size at start and end of experiment *Figures in parentheses represent ranges

Groups	Number of fed bed bugs at start of experiment	Number of alive bed bugs on day 54	Fold change
Control	306	662	2.16
4 hours	298	27	-11.04
12 hours	191	58	-3.29
24 hours	319	174	-1.83
36 hours	208	89	-2.34
48 hours	206	167	-1.23
60 hours	192	132	-1.46
72 hours	197	217	1.10
84 hours	182	209	1.15
96 hours	174	249	1.43
Median for 4-96 hours	197 (174-319)*	167 (27-249)*	-1.46 (-11.04-1.43)*

**Figure 1:**
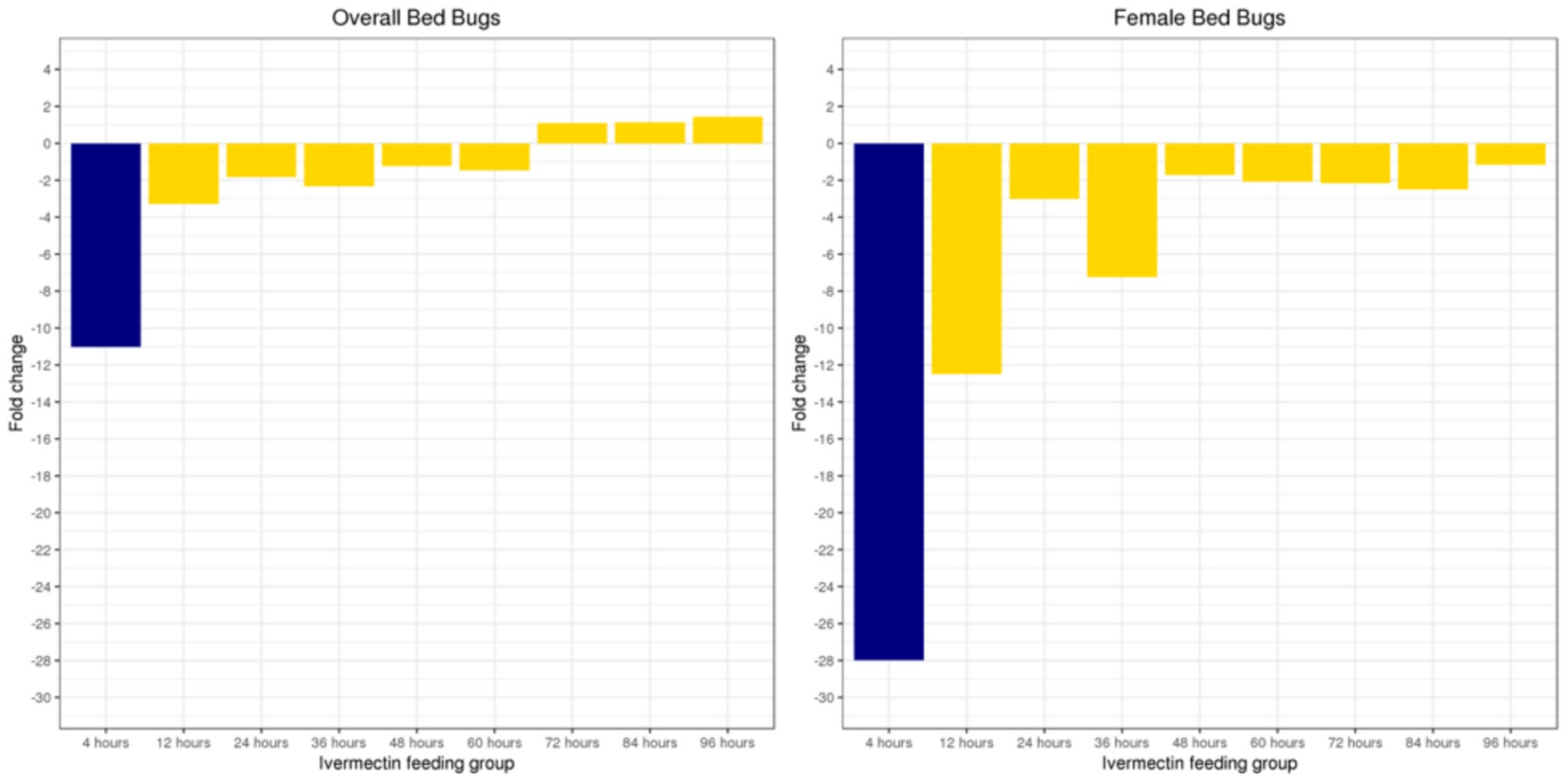
The fold change in the overall and female bed bug population from start to day 54 of experiment

The fold change in the alive bed bug population size was significantly different for post-ivermectin feeding times (median: -1.46; range: -11.04- 1.43) when compared to controls (2.16, p: 0.004, Figure 1).

The 4-60-hour post-ivermectin feeding groups were the most impacted whereas the 4-hour post-ivermectin feeding group had the largest decrease in population with a fold change of -11.04. In contrast, all 72-96-hour post-ivermectin feeding groups experienced a net increase in population size with fold changes ranging from 1.10 to 1.43. Further investigation revealed that the expected number of overall alive bed bugs was estimated to increase by 1.9% (95% CI: 1.8-2.2%) for every hour after the original ivermectin feeding (p: <0.001).

Additionally, we observed at day 14 of post-ivermectin ingestion that the 4-72-hour post-ivermectin feeding groups had bed bugs that had either not digested their blood meals or had only partially digested their blood meals. The control group and the 84-96-hour post-ivermectin groups had largely digested their blood meals. There were no molts at day 14 in the 4-36-hour post-ivermectin fed groups.

All post-ivermectin feeding groups showed significantly higher incapacitation rates (range: 89-100% vs. 12% in controls) and increased mortality (range 27-97% vs. 6% in controls) on day 7 (p: <0.001, Table 2). At day 14 of post-ivermectin ingestion, there was significantly higher bed bug mortality at post-feeding hour 4 (54%) and hour 12 (46%) compared to controls (22%, p: <0.001). Observed mortality decreased between day 7 and 14 in all post-ivermectin feeding groups except for the 48-hour group, indicating that some insects which appeared to be dead were able to recover at least some measure of their life functions.

**Table 2:** Overall bed bug incapacitation on day 7 and mortality rates on day 7 and day 14 after ivermectin ingestion

Groups	Incapacitation rate on day 7, %	P-value compared to control	Mortality rate on day 7, %	P-value compared to control	Mortality rate on day 14, %	P-value compared to control
Control	12		6		22	
4-hour	100	<0.001	97	<0.001	54	<0.001
12-hour	100	<0.001	80	<0.001	46	<0.001
24-hour	89	<0.001	48	<0.001	33	0.11
36-hour	100	<0.001	43	<0.001	29	0.33
48-hour	93	<0.001	31	<0.001	34	0.083
60-hour	93	<0.001	30	<0.001	29	0.33
72-hour	100	<0.001	37	<0.001	31	0.26
84-hour	100	<0.001	37	<0.001	24	0.87
96-hour	100	<0.001	27	<0.001	24	0.87

The number of alive adult females on day 54 was also significantly lower compared to the number of initially fed females among the post-ivermectin groups where the median fold change was -2.50 (range: -28.00 to -1.16, Table 3). Furthermore, the number of alive female bed bugs was estimated to increase by 1.9% (95% CI: 1.2-2.8%) with each hour after the original ivermectin feeding (p: <0.001).

**Table 3:** Female bed bug population size at start of experiment and 54 days after ivermectin ingestion *Figures in parentheses represent ranges

Groups	Number of fed bed bugs at start of experiment	Number of alive bed bugs on day 54	Fold change
Control	39	40	1.03
4 hours	28	1	-28.00
12 hours	25	2	-12.50
24 hours	27	9	-3.00
36 hours	29	4	-7.25
48 hours	19	11	-1.73
60 hours	25	12	-2.08
72 hours	26	12	-2.17
84 hours	30	12	-2.50
96 hours	22	19	-1.16
Median for 4-96 hours	26 (19-30)*	11 (1-19)*	-2.50 (-28.00 to -1.16)*

Table 4 summarizes the incapacitation rates for adult female C. lectularius 14 days after ivermectin ingestion. Adult female bed bugs in groups 4, 12, 24, 72 hours post-ivermectin ingestion had significantly higher incapacitation rates (≥28%) compared to controls (0%, p: <0.001).

**Table 4:** Female bed bugs incapacitation on day 14 after ivermectin ingestion *Figures in parentheses represent the absolute numbers for incapacitated bed bugs/totals for the respective group

Groups	Incapacitation rate on day 14	P-value compared to control
Control	0% (0/39)*	
4 hours	36% (10/28)*	<0.001
12 hours	28% (7/25)*	<0.001
24 hours	41% (11/27)*	<0.001
36 hours	4% (1/29)*	0.43
48 hours	0% (0/19)*	1.00
60 hours	0% (0/25)*	1.00
72 hours	31% (8/26)*	<0.001
84 hours	3% (1/30)*	0.44
96 hours	5% (1/22)*	0.36

**Figure 2:**
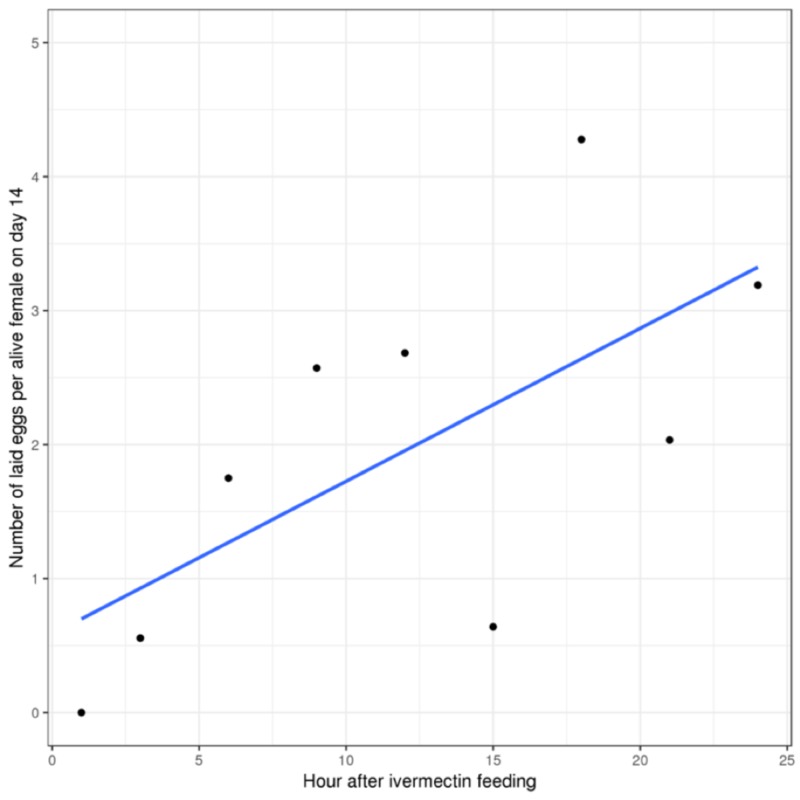
Number of eggs laid per alive female bed bug per hour on day 14 after ivermectin injection

Ivermectin was also associated with a significant reduction in the number of eggs laid per female in all the post-ivermectin feeding groups (median: 2.03, range: 0.00-4.28), when compared to the control group (10.74, p: <0.001, Table 5). However, further investigation revealed that the number of eggs laid per female was estimated to increase by 0.11 (-0.00-0.23) eggs every four hours (p: 0.052, Figure 2).

**Table 5:** The number of Cimex lectularius L. eggs laid on day 14 after ivermectin ingestion *Figures in parentheses represent ranges

Groups	Total number of eggs	Number of alive adult female bed bugs	Eggs laid per alive adult female bed bugs
Control	419	39	10.74
4 hours	0	18	0.00
12 hours	10	18	0.56
24 hours	28	16	1.75
36 hours	72	28	2.57
48 hours	51	19	2.68
60 hours	16	25	0.64
72 hours	77	18	4.28
84 hours	59	29	2.04
96 hours	67	21	3.19
Median for 4-96 hours	51 (0-77)*	19 (16-29)*	2.03 (0.00-4.28)*

Table 6 shows the whole blood ivermectin levels measured in the subjects. Ivermectin levels were below the limits of detection at hour 48 for subject 1 and 36 for subject 2. Table 7 shows ivermectin levels in C. lectulariusat different times post-ivermectin feeding. We were able to detect ivermectin levels at 0.2 ng/insect in a male killed immediately after it fed at hour 72, a mean 0.16 ng/insect in males killed immediately after feeding at hour 60, and a mean 0.13 ng/insect in males killed immediately after feeding at hour 48. We were unable to detect ivermectin four days after in a female fed at hour 4, two days after in females fed at hour 48, 12 days after in males fed at hour 48, and 12 days after in males fed at hour 60.

**Table 6:** Whole blood ivermectin levels in study subjects *Below the limits of detection ng/mL: nanogram/milliliter

Hour post-ivermectin ingestion	Subject 1 whole blood ivermectin level, ng/mL	Subject 2 whole blood ivermectin level, ng/mL
4 hours	38.1	17.7
12 hours	5.8	2.6
24 hours	1.9	2.0
36 hours	0.6	*
48 hours	*	*

**Table 7:** Ivermectin concentration in Cimex lectularius L. *Figures in parentheses represent the number of insects ng: nanogram

Life stage of bed bugs	Groups	Days after the insect was killed post-feed	Mean ivermectin per bed bug, ng
Adult female (1)*	4 hours	4	0
Adult male (1)*	72 hours	0	0.2
Adult female (2)*	48 hours	2	0
Adult male (3)*	60 hours	0	0.16
Adult male (3)*	48 hours	0	0.13
Adult male (3)*	48 hours	12	0
Adult male (4)*	60 hours	12	0

## Discussion

The data show that ivermectin is able to cause long-term harm to bed bug populations after the insects had fed once on blood taken from persons 4-96 hours after they had consumed 0.2 mg/kg of ivermectin. None of the feeding groups experienced 100% mortality in their bed bug populations, but the 4-hour feeding group went from 298 live bed bugs down to 27 at 54 days. It remains unclear if the groups with the highest mortality (e.g., 4 hours post-ivermectin ingestion) would have eventually eliminated their bed bug populations or the population would eventually rebound. Previous laboratory research has shown that severely affected insects may never recover to normal function [20-22]. It is also likely that these severely affected insects would not be able to refeed and may eventually die of starvation [21,22]. The group with Insects that fed on blood obtained ≥72 hours post-ivermectin consumption had higher total numbers of insects at 54 days than when the experiment started, although the differences were significantly less compared to the controls. These results are consistent with previous work showing ivermectin has a dose-responsive effect on bed bugs.

The HPLC/MS data showed that whole blood ivermectin levels were below the limits of our detection (~0.05 ng/mL) after about 36 hours of taking the drugs. Previous research in which ivermectin was added to blood samples in the laboratory shows that about 5-20 ng/mL of ivermectin was required for significant long-term bed bug toxicity [20-22]. The reductions in fecundity and overall size of the bed bug populations in the 48-96-hour groups suggest that ivermectin may have metabolites that are toxic to C. lectularius. Additionally, we were not able to measure ivermectin levels in bed bugs ≥2 days post-ivermectin ingestion but were able to measure ivermectin levels in insects killed on the day of the feeding. This could mean that C. lectularius is able to excrete or metabolize ivermectin over time, the levels of ivermectin in the insects are below the limits of our detection, or that ivermectin metabolites in human blood (which were not measured in our assay) are causing toxic effects. The latter hypothesis is unlikely to fully explain our results because, as has been previously mentioned, blood samples spiked with ivermectin also cause long-term bed bug morbidity and mortality [22]. Additionally, using HPLC/MS, ivermectin has been detected in C. lectularius for up to a month after feeding on blood meals spiked with ivermectin in the laboratory (unpublished data). Despite not being able to detect ivermectin in the 4-hour feeding group four days after the blood meal and, in the 48-hour feeding group, two days after the blood meal, these groups suffered significant long-term reductions in fecundity and overall population size compared to controls.

The results suggest that a single dose of ivermectin at 0.2 mg/kg will be unlikely to be able to eliminate a bed bug infestation when used in isolation. However, the use of ivermectin along with traditional integrated pest management (IPM) practices could be useful in large, well- established, or difficult-to-treat infestations. Alternatively, higher or more frequent doses of ivermectin could have greater utility in controlling an infestation. Single doses of 2 mg/kg of ivermectin in humans, a 10-fold higher dose than used in our study, was shown to be safe and well-tolerated [26]. Additionally, 0.2 mg/kg of ivermectin given on days 1, 2, 8, 9, 15, 22, and 29 is used clinically to treat human crusted scabies [30]. A clinical trial using ivermectin plus IPM compared to IPM-only may be warranted and could help determine if ivermectin could be beneficial in managing bed bug infestations over current techniques.

Limitations

Insects in our experiment were kept under laboratory conditions, which may not replicate all the challenges bed bugs encounter in their natural environment, including seeking refuge (harborage), molting, and re-feed. Ivermectin is known to affect all these three activities [20-22]. Combining the incapacitation rates of the bed bugs from both subjects may have limited the ability to detect differences in incapacitation rates that could exclusively exist in infects fed from a particular subject. Only two subjects were used in the experiments. However, the study did not try to assess the effectiveness of ivermectin in harming bed bugs in individual subjects.

## Conclusions

The study design allowed for efficient investigation of longitudinal ivermectin toxicity on bed bug incapacitation, fecundity, and population size. Overall, ivermectin significantly reduced the overall and female bed bug population, increased mortality and incapacitation rates, and reduced the number of eggs laid per female. Evidence suggests that the earlier bed bugs ingest host blood samples after ivermectin ingestion, the greater the toxic impact. Further research is warranted to understand the full impact of ivermectin toxicity on bed bugs over time and to determine whether the drug can be used clinically to aid in the elimination of bed bug infestations.
